# Supplementary material for: Testing Explant Sources, Culture Media, and Light Conditions for the Improvement of Organogenesis in Pinus ponderosa (P. Lawson and C. Lawson)
Source: Plants (Basel). 2023 Feb 14;12(4):850. doi: 10.3390/plants12040850 (PMC9966710; doi:10.3390/plants12040850)
Supplement: Supplementary file 1 [file plants-12-00850-s001.zip › plants-2205359-supplementary.pdf]

Supplementary materials

Table S1. Statistical analysis for the survival (%) showed in explants of *Pinus ponderosa* per 6-benzyladenine (BA) (4.4, 22 and 44 μM) and cultured media (LP or half LP macronutrients) (Quoirin and Lepoivre [24], modified by Aitken-Christie et al. [25]).

Source	df	Survival (%)	
		**X^2 Test**	***p*-Value**
BA concentration (B)	2	600.83	≤0.05*
Culture medium (C)	1	608.99.	>0.05 n.s
B x C	1	597.87	>0.05 n.s

*Significantly different at $p<0.05$, n.s Non-significant at $p<0.05$, df Degrees of freedom.

Table S2. Statistical analysis for the survival (%) showed in explants of *Pinus ponderosa* per explant type (cotyledons or whole zygotic embryos) and sterilization protocol [5% commercial bleach and 10% H_2O_2 (v/v)].

Source	df	Survival (%)	
		**X^2 Test**	***p*-Value**
Explant type (E)	1	596.21	≤0.05*
Sterilization protocol (S)	1	593.11	>0.05 n.s
E x S	1	592.96	>0.05 n.s

*Significantly different at $p<0.05$, n.s Non-significant at $p<0.05$, df Degrees of freedom.

Table S3. Statistical analysis for the explants forming shoots (EFS) (%) showed in explants of *Pinus ponderosa* per 6-benzyladenine (BA) (4.4, 22 and 44 µM), culture media (LP or half LP macronutrients) (Quoirin and Lepoivre [24], modified by Aitken-Christie et al. [25]), explant type (cotyledons or whole zygotic embryos) and sterilization protocol [5% commercial bleach and 10% H_2O_2 (v/v)].

Source	df	EFS (%)	
		**X^2 Test**	***p*-Value**
BA concentration (B)	2	496.36	>0.05 n.s
Cultured media (C)	1	498.04	≤0.05*
Explant type (E)	1	534.93	≤0.05*
Sterilization protocol (S)	1	534.93	>0.05 n.s
E x S	1	496.36	>0.05 n.s
E x C	1	454.59	≤0.05*
C x S	1	453.84	>0.05 n.s
B x E	2	453.54	>0.05 n.s
B x S	2	452.56	>0.05 n.s
B x C	2	452.18	>0.05 n.s
E x C x S	1	448.81	>0.05 n.s
B x E x S	2	448.19	>0.05 n.s
B x C x E	2	443.74	>0.05 n.s
B x C x S	2	441.34	>0.05 n.s
B x C x E x S	2	436.19	>0.05 n.s

*Significantly different at $p<0.05$, n.s Non-significant at $p<0.05$, df Degrees of freedom.

Table S4. Statistical analysis for the survival (%) showed in explants of *Pinus ponderosa* per cytokinin type and light treatment cultured in half LP macronutrients (Quoirin and Lepoivre [24], modified by Aitken-Christie et al. [25]).

Source	df	Survival (%)	
		**X^2 Test**	***p*-Value**
Cytokinin type (C)	1	29.206	≤0.05*
Light treatment (C)	3	21.311	≤0.05*
C x L	3	21.311	>0.05 [n.s]

*Significantly different at $p<0.05$, [n.s] Non-significant at $p<0.05$, df Degrees of freedom.

Table S5. Statistical analysis for the explants forming shoots (EFS) (%) showed in explants of *Pinus ponderosa* cultured in half LP macronutrients (Quoirin and Lepoivre [24], modified by Aitken-Christie et al. [25]) supplemented with 6-benzyladenine (BA) and *meta*-Topolin (*m*-T) according to light treatment.

Source	df	EFS (%)	
		**X^2 Test**	***p*-Value**
Cytokinin type (C)	1	393.51	>0.05 [n.s]
Light treatment (C)	3	392.40	>0.05 [n.s]
C x L	3	391.23	>0.05 [n.s]

[n.s] Non-significant at $p<0.05$, df Degrees of freedom.

Table S6. ANOVA for total number of shoots produced per initial explant (NS/E) showed in explants of *Pinus ponderosa* cultured in half LP macronutrients (Quoirin and Lepoivre [24], modified by Aitken-Christie et al. [25]) supplemented with 6-benzyladenine (BA) and *meta*-Topolin (*m*-T) (13.1 μM) according to light treatment.

Source	df	NS/E	
		***F* Test**	***p*-Value**
Cytokinin type (C)	1	0.31	>0.05 n.s
Light treatment (C)	3	1.00	>0.05 n.s
C x L	3	1.07	>0.05 n.s

*Significantly different at p<0.05, n.s Non-significant at p<0.05, df Degrees of freedom.

Table S7. ANOVA for percentage of shoots elongated enough to be rooted (PSR) (%) showed in explants of *Pinus ponderosa* cultured in half LP macronutrients (Quoirin and Lepoivre [24], modified by Aitken-Christie et al. [25]) supplemented with 6-benzyladenine (BA) and *meta*-Topolin (*m*-T) (13.1 μM) according to light treatment.

Source	df	PSR (%)	
		***F* Test**	***p*-Value**
Cytokinin type (C)	1	0.02	>0.05 n.s
Light treatment (C)	3	3.94	≤0.05*
C x L	3	0.21	>0.05 n.s

*Significantly different at p<0.05, n.s Non-significant at p<0.05, df Degrees of freedom.

Table S8. Statistical analysis for root induction (%), number root per explant and length of longest root showed in explants of *Pinus ponderosa* cultured in half LP macronutrients (Quoirin and Lepoivre [24], modified by Aitken-Christie et al. [25]) according to light treatment.

Source	df	Root induction		Nº root /explant		Length of longest root	
		**X^2 Test**	***p*-Value**	***F* Test**	***p*-Value**	***F* Test**	***p*-Value**
Light treatment (L)	3	132.79	>0.05 [n.s]	0.66	>0.05 [n.s]	0.42	>0.05 [n.s]

[n.s] Non-significant at $p<0.05$, df Degrees of freedom.
